# Comparative outcomes of internal fixation versus prosthetic reconstruction in the treatment of proximal femoral metastases: a systematic review and meta-analysis

**DOI:** 10.1530/EOR-2024-0131

**Published:** 2025-11-03

**Authors:** Ya-Shih Lai, Shu-Han Chuang, Yi-Jie Kuo, Shun-Jen Cheng, Yu-Pin Chen

**Affiliations:** ^1^ Division of General Practice, Department of Education, Taoyuan General Hospital, Ministry of Health and Welfare, Taoyuan, Taiwan; ^2^ Department of Ophthalmology, Changhua Christian Hospital, Changhua, Taiwan; ^3^ Department of Orthopedics, Wan Fang Hospital, Taipei Medical University, Taipei, Taiwan; ^4^ Department of Orthopedics, School of Medicine, College of Medicine, Taipei Medical University, Taipei, Taiwan

**Keywords:** internal fixation, prosthesis, reconstruction, femoral metastases, meta-analysis

## Abstract

**Background:**

- Metastatic disease frequently causes pathological fractures, particularly in the proximal femur, significantly impacting patient prognosis and quality of life. With the advancements in cancer treatment leading to longer survival, there is a pressing need to evaluate the outcomes of surgical interventions aimed at managing proximal femoral metastases. This study compares the outcomes of internal fixation (IF) versus prosthesis (P) in the treatment of proximal femoral metastases, focusing on survival times, complication rates, functional outcomes, and reoperation rates.

**Method:**

- A systematic review and meta-analysis were conducted, searching PubMed, Embase, and Cochrane databases for studies published up to December 31, 2023. The PRISMA guidelines were followed. Studies comparing IF and P for proximal femoral metastases were included. Data on survival times, blood loss, reoperation rates, and functional scores were extracted and analyzed using the forest plot technique and inverse variance method. The quality of included studies was assessed using the Newcastle–Ottawa scale.

**Results:**

- In total, 19 studies (16 retrospective and three prospective) involving 4,126 patients were included. The P group demonstrated significantly longer survival times compared with the IF group, with no significant difference in complication and reoperation rates between the two methods. However, IF was associated with shorter operative times and less blood loss.

**Conclusion:**

- P may provide superior long-term functional outcomes and extended survival compared with IF, with similar rates of complications and reoperations. However, selection bias – where healthier patients with better baseline physiology are more likely to undergo prosthetic reconstruction – significantly impacts the interpretation of these findings, underscoring the need for further prospective studies.

## Introduction

Metastatic disease often leads to pathological fractures (1), and in 2023, an estimated 1,958,310 new cancer cases and 609,820 cancer-related deaths were projected in the USA (2). Bone metastases will affect around 5% of these patients, involving various cancer types, including lung, breast, prostate, multiple myeloma, and lymphoma (3). Among these cases, approximately 4.4% present with pathologic fractures (4, 5), and the femur is the most common site of metastasis (6, 7). Given that the femur is a weight-bearing bone, even minor injuries can weaken its structure (8). The proximal femur experiences some of the body’s highest loads (9), and metastasis can compromise the architecture and strength of cortical and trabecular bone (7). Pathologic fractures significantly impact prognosis, affecting survival time, with almost half of the individuals experiencing symptoms, such as pain, lumps, limited joint activity, and restrictions in weight-bearing, leading to disability in daily activities (6, 10). Therefore, evaluating and managing femoral pathologic fractures is crucial.

Patients suspected of having pathologic fractures should undergo a comprehensive assessment by a multidisciplinary team. This assessment, which includes a thorough examination of their medical history, imaging studies, and clinical presentation, should precede the development of a therapeutic plan (11). Mirel’s criteria, a widely recognized prognostic tool with 91% sensitivity, assess the risk of pathological fractures in bone lesions (12). This classification system, considering factors such as site, location, matrix, and pain presence, is valuable for evaluating patients with metastatic involvement in long bones. Surgical intervention for pathologic fractures is commonly considered a palliative procedure, aiming to provide pain relief and enhance the stability of the bone structure. However, recent advances in chemotherapy and molecular targeted therapeutics offer longer patient life expectancy and increased attention to life quality, making surgery more crucial in contemporary medical practice (13, 14, 15).

Current surgical options for managing proximal femoral bone metastasis include internal fixation (IF) versus prosthesis (P) procedures (6, 11, 13, 14, 16). Controversy exists as numerous studies compare outcomes, some advocate including survival time, blood loss, functional improvement, and complication rates between these two options. Some advocate for IF (17, 18, 19), citing its lower cost, simplicity, and (20, 21, 22) suitable for patients with a short life expectancy. There is no consensus on which is better (20, 21, 22, 23, 24). Various complex factors influence outcomes and management, leading to a lack of consensus on the most suitable surgical option for every patient (23, 24).

Therefore, this systematic review and meta-analysis aimed to examine and compare outcomes, such as survival times, blood loss, complications, reoperation rates, and functional scores, associated with both IF and P for the management of proximal femoral bone metastasis.

## Materials and methods

### Study design

This meta-analysis adhered to the Preferred Reporting Items for Systematic Reviews and Meta-Analyses (PRISMA) guidelines and 2020 PRISMA checklist (25, 26). This study was registered in an international database of prospectively registered systematic reviews. The entire process, including literature search, study selection, data extraction, quality assessment, and statistical analysis, was conducted independently by two authors. Any disagreements were resolved through discussion with a third author.

### Search strategy

For the systematic review and meta-analysis, we searched PubMed, Embase, and Cochrane for relevant articles published up to December 31, 2023. The search strategy combined terms such as “proximal,” “femur,” and “metastasis,” following methodology filter recommended by the Harvard Countway Library (https://guides.library.harvard.edu/meta-analysis), the study design was considered in the search strategy. A detailed search term list can be found in the Supplementary materials (Supplementary_File_Search_Strategy (see the section on Supplementary materials given at the end of the article)). No date or language restrictions were applied, except for final inclusion of English articles.

### Eligibility criteria

Following the completion of the searches, we eliminated duplicate articles. Inclusion criteria comprised studies meeting the following conditions: i) relevant studies reporting surgical outcomes for proximal femoral pathologic fractures, ii) presented in English, and iii) original journal articles (excluding review articles, letters, or those presented in conference, proceedings, seminar, symposium, and workshop formats). We excluded review articles, letters, conference abstracts, and those not specifically comparing IF and P.

### Data extraction and measurements

Two authors independently extracted data using a well-designed table that incorporated information, such as authors, publication year, country, total participants, gender, age, study design, and follow-up duration. Primary outcomes included overall patient survival, while secondary outcomes included reoperation rates, complications, operative duration, blood loss, and MSTS (Musculoskeletal Tumor Society) scores (27).

### Quality assessment

The quality assessment of the included studies was conducted independently by two reviewers, using the Newcastle–Ottawa scale (NOS) with a scoring system covering selection (0–4 points), comparability (0–2 points), and outcome (0–3 points), totaling a maximum of 9 points (28). Any disparities in assessments were resolved through discussion with the corresponding author, with results summarized in Supplementary Table 2.

### Statistical analysis

The data extracted from our research underwent analysis using the Comprehensive Meta-Analysis software, version 3.0, developed by Biostat, NJ, specifically for meta-analysis. We treated survival, operative time, blood loss, and MSTS scores as continuous variables, reporting mean differences (MDs) with 95% confidence intervals (CIs). Reoperation and complication rates were treated as dichotomous variables, reporting odds ratios with 95% CIs. We used a random-effects model rather than a fixed-effect model to account for potential between-study heterogeneity, which was assessed using the chi-squared test, Cochrane Q, and *I*^2^. For publication bias, we employed funnel plots and Egger’s test, followed by trim-and-fill if bias was suspected. When applicable, pretest and posttest scores were compared with evaluate the effect of interventions (29). The change scores, representing the difference between posttest and pretest scores, were calculated as follows:

Change score = posttest score − pretest score.

The standard deviation (SD) of the change scores was calculated using the following formula:

$${\text{SD}}_{\text{change}}\;=\sqrt{SD_{\text{pre}}^{2}\;+\;SD_{\text{post}}^{2}\;‐2\;\times \;r\;\times \;SD_{\text{pre}}^{2}\;SD_{\text{post}}^{2}},$$

where $SD_{\text{pre}}^{2}$ and $SD_{\text{post}}^{2}$ are the SDs of the pretest and posttest scores, respectively, and r is the assumed correlation (set to 0.5 based on literature recommendations) (30, 31, 32).

In this meta-analysis, we calculated pooled estimates using the DerSimonian and Laird random-effects approach to accommodate varying effect sizes across studies (33). Heterogeneity was quantified via the Q statistic (with a significance threshold of *P* < 0.10) and *I*^2^. When *I*^2^ >50%, we interpreted it as at least moderate heterogeneity. Egger’s regression test was used to statistically evaluate small-study effects, and funnel plots were visually inspected. If publication bias was indicated, the trim-and-fill method by Duval and Tweedie was applied to estimate the impact of potentially missing studies on the overall effect size (34, 35).

## Result

### Study selection

Initially, 1,231 articles were identified (354 PubMed, 857 Embase, and 20 Cochrane). After removing 285 duplicates, 946 articles remained. Following the screening process based on the inclusion criteria, 51 articles were eligible for full-text assessment. Among these, 33 were excluded as they did not meet the criteria of being comparative studies specifically focusing on surgery involving IF versus P procedures. Ultimately, 18 articles were included for the review. In addition, one article was included through citation searching, bringing the total to 19 articles included in the analysis (17, 36, 37, 38, 39, 40, 41, 42, 43, 44, 45, 46, 47, 48, 49, 50, 51, 52).

**Figure 1:**
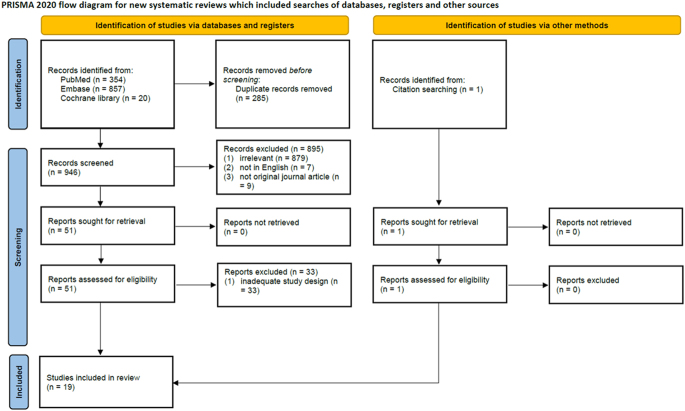
PRISMA flow diagram for study selection. This figure illustrates the study selection process using the PRISMA (preferred reporting items for systematic reviews and meta-analyses) framework. It shows the identification, screening, eligibility, and inclusion of studies. Initially, 1,231 articles were identified, and after the removal of duplicates and applying eligibility criteria, 19 studies were included in the final meta-analysis.

Figure 1 depicts the selection process in detail, including identification of records, removal of duplicates, screening steps, and final inclusion decisions.

Supplementary Table 1 presents a comprehensive compilation of research studies focused on the surgical management of metastatic bone tumors in the proximal femur. Of these, 16 studies were retrospective, and three studies were prospective. The data spans multiple years, ranging from 2001 to 2023, providing detailed information on sample sizes (ranging from *n* = 20–1,497), study designs, target groups, follow-up times, and various outcomes. The most common primary cancer is variable because the case number is small in each study, and it originates from breast, following by lung, prostate, kidney and multiple myeloma. These outcomes include survival time, reoperation rate, complication rate, operative time, blood loss, and MSTS score. Surgery methods are classified as IF and P. IF includes fixation with intramedullary nail, dynamic hip screw, cannulated screw, cephalomedullary or reconstruction nail. P includes partial or total replacement with cemented or uncemented prosthesis, hemiarthroplasty and tumor endoprosthetic reconstruction. These data serve as a valuable resource, offering insights into diverse global surgical strategies for managing metastatic bone tumors in the proximal femur and providing a comprehensive overview of the current state of research in this field. The quality score ranged from 0 to 9 from NOS.

### Survival time

**Figure 2:**
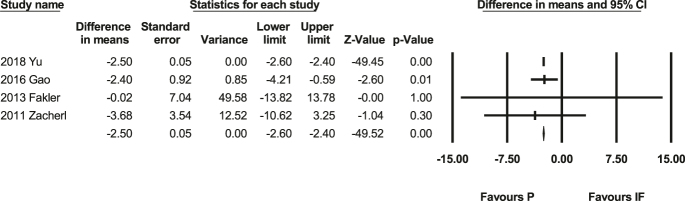
Forest plot of survival time (41, 46, 48, 50). This forest plot compares the survival time between patients undergoing IF and prosthetic reconstruction (P). The pooled MD and 95% CI favor prosthetic reconstruction (P), indicating a longer survival time compared with IF. Statistical heterogeneity was minimal (*I*^2^ = 0%).

The meta-analysis of survival time includes four studies (41, 46, 48, 50) with 286 people. The difference in means is negative (MD: −2.50, *P* < 0.001; 95% CI: −2.6, −2.4, Fig. 2), representing favors P. In other words, the overall survival outcomes suggest that the prosthesis group exhibits a longer survival rate compared with the IF group. The heterogeneity test showed Q-value: 0.25, *P* = 0.97, *I*-squared: 0, Supplementary Fig. 1. It also indicates a lack of significant publication bias, as evidenced by a non-significant Egger’s test (*P* = 0.85).

### Function

The MSTS score is a commonly used system for assessing the functional status of patients following musculoskeletal tumor surgery. It considers various parameters related to pain, function, emotional acceptance, and support. There are three studies that include MSTS score (37, 41, 42).

**Figure 3:**
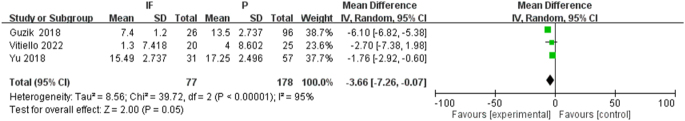
Forest plot of MSTS functional scores (37, 41, 42). This forest plot evaluates the functional outcomes (MSTS scores) of patients treated with IF versus prosthetic reconstruction (P). The results favor prosthetic reconstruction (P), showing significantly higher MSTS scores. High heterogeneity (*I*^2^ = 95%) was observed due to varying follow-up durations among included studies.

The meta-analysis of survival time includes three studies with 255 people. The difference in means is negative (MD: −3.66, *P* < 0.001; 95% CI: −7.26, −0.07, Fig. 3), representing favors *P*. In other words, the overall function suggest that the prosthesis group exhibits a better performance compared with the IF group. This forest plot demonstrates significant heterogeneity (*I*^2^ = 95%) among the included studies. This high degree of variability is primarily attributed to the inclusion of only three studies in the analysis, each with differing follow-up periods for the MSTS (Musculoskeletal Tumor Society) score assessments. The follow-up intervals varied among the studies, with assessments at weeks and months. Such variation in the timing of outcome measurements contributes to the observed heterogeneity by influencing the consistency of the results across the studies.

### Reoperation rate

**Figure 4:**
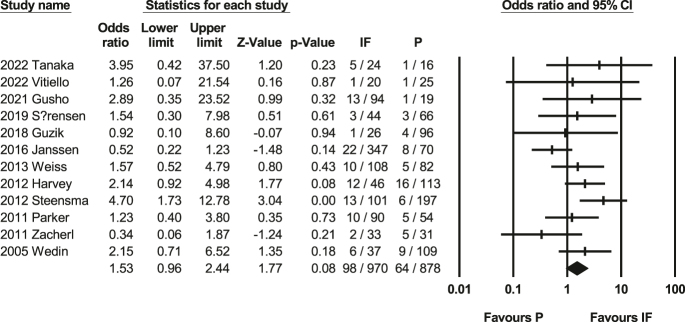
Forest plot of reoperation rates (17, 37, 38, 40, 42, 45, 47, 49, 50, 51, 52). This forest plot compares reoperation rates between IF and prosthetic reconstruction (P). No statistically significant difference was found between the two methods. Heterogeneity was moderate (*I*^2^ = 32%), and Egger’s test indicated no publication bias.

A meta-analysis calculating the odds ratio of the reoperation rate, including 12 studies (17, 37, 38, 40, 42, 45, 47, 49, 50, 51, 52) with 1,748 people, was conducted. It reveals that there is not statistically significant (*P* = 0.08, 95% CI: 0.96–2.44, Fig. 4) between the IF group and P group. Heterogeneity test showed *Q*-value: 16.24, *P*-value = 0.13, and *I*-squared: 32.27, Supplementary Fig. 2, which suggests a moderate level of heterogeneity. The assessment of reoperation rates indicates a lack of significant publication bias, as reflected by a non-significant Egger’s test (*P* = 0.98).

### Complication rate

**Figure 5:**
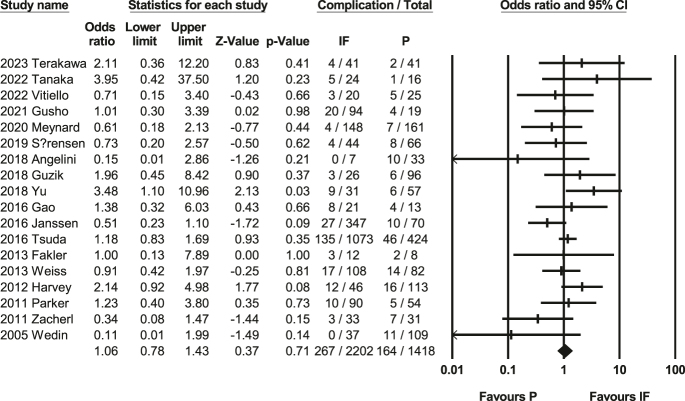
Forest plot of complication rates (17, 36, 37, 38, 39, 40, 41, 42, 43, 44, 45, 46, 47, 48, 49, 50, 51, 52). This forest plot compares complication rates between IF and prosthetic reconstruction (P). The meta-analysis showed no significant difference between the two groups. Heterogeneity was low (*I*^2^ = 19%), and no evidence of publication bias was detected.

A meta-analysis of the complication rates associated with 18 studies (17, 36, 37, 38, 39, 40, 41, 42, 43, 44, 45, 46, 47, 48, 49, 50, 51, 52) with 3,440 people was conducted, revealing a non-significant difference (*P* = 0.71, 95% CI: 0.78–1.43, Fig. 5) between IF and prosthetic interventions. Heterogeneity test showed *Q*-value: 21.20, *P*-value: 0.22, *I*-squared: 19.82, and Supplementary caution due to inherent limitations in the included studies, including potential selection bias Fig. 3. The assessment of complication rates indicates a lack of significant publication bias, as reflected by a non-significant Egger’s test (*P* = 0.49).

### Operation time

A meta-analysis of the operative time associated with IF and P interventions across nine studies (17, 36, 37, 38, 40, 41, 45, 46, 48) with 1,443 people revealed significantly shorter operative times for IF compared with prosthetic reconstruction. This was shown by a MD of 51.49 min (95% CI: −70.99 to −32.00, *P* < 0.001, Supplementary Fig. 4). However, it is noteworthy that two studies, specifically the 2019 study by Sørensen (*P* = 0.08) (41) and the 2013 study by Fakler (*P* = 0.07) (49), did not align with this trend. Heterogeneity test showed *Q*-value: 128.16, *P*-value: 0.00, and *I*-squared: 93.76, Supplementary Fig. 5, which reported a high level of heterogeneity in the operative time outcomes among the included studies. Notably, Egger’s test showed a significant bias with a two-tailed *P*-value of 0.03. To address this bias, Duval and Tweedie’s trim and fill method were employed, identifying and adjusting for three additional studies. The studies resulted in a point estimate showing a decrease in operative time by about 65 units in random effect. These findings underscore the variability in operative time with attention to potential publication bias and adjustments to enhance the reliability of the meta-analysis results.

### Blood loss

A meta-analysis of the blood loss associated with IF and P interventions across seven studies (17, 36, 38, 40, 41, 45, 46) with 1,378 people was conducted. It revealed that blood loss favoring IF as being significantly lower (*P* = 0.00, 95% CI: −340.69 ∼ −209.96, Supplementary Fig. 6) by MD of 275.33. Heterogeneity test showed *Q*-value: 22.21, *P*-value: 0.00, *I*-squared: 72.99, Supplementary Fig. 7, as a moderate level of heterogeneity. In addition, the assessment for publication bias using Egger’s test (*P* = 0.77) suggested that there is no substantial bias in the reported results. This underscores the reliability of the meta-analysis findings, reinforcing the conclusion that IF is associated with a significantly lower blood loss when compared with alternative interventions.

## Discussion

In our meta-analysis of the included studies, it was observed that survival time tends to be longer and function is better with P compared with IF. The rates of reoperation and complications between the two methods showed no significant difference. IF was associated with shorter operative times and less blood loss compared with prosthetic reconstruction.

Although our study revealed that patients undergoing P tend to have longer survival times, this result should be interpreted cautiously due to the potential influence of selection bias. Surgeons often choose IF for patients in poorer health, as it involves shorter operative times and reduced blood loss (52), while healthier patients with longer expected survival and greater functional demands are more likely to receive prosthetic reconstruction. This tendency can artificially inflate the observed survival advantage of P. For example, Fakler *et al.* (49) showed that patients undergoing P had better preoperative health status, including higher Karnofsky performance scores and greater ambulatory capacity, compared with those receiving IF, while Steensma *et al.* (23) reported a higher proportion of patients undergoing P with favorable functional scores. These baseline differences likely explain the observed survival and functional advantages in the prosthetic reconstruction group. These selection biases show the limitations of current studies and emphasize the need for future research to control for baseline differences and use prospective designs for a clearer comparison between surgical methods. These adjustments are especially important considering that patients’ preoperative condition and tumor biology may strongly influence surgical decision-making and outcomes.

Age is also an influential factor for survival. Based on the survival outcomes included in the four studies, the average age for the IF group is 67.15, while for the P group, it is 62.9. It is evident that the IF group is significantly older. Fakler *et al.* (49) reported a notable age difference between the two surgical groups (IF group: 61.9 years, P group: 73.8 years, *P* < 0.05) in a cohort study, finding that the older IF group showed a lower survival time (2 vs 4.5 months, *P* = 0.58). On the other hand, the sites and types of primary cancer may influence the outcome of survival time (53). Zacherl *et al.* acknowledged that the type of primary cancer – originating from the breast, prostate, lung, kidney, or colon – may affect the rate of bone metastases, thereby indirectly influencing survival time (52). Cappellari *et al.* (54) reported that patients with solitary or oligometastatic lesions had significantly better survival than those with multiple metastases (*P* < 0.0001). They also found that resection with prosthesis was associated with higher 2- and 4-year survival rates compared with intramedullary nailing (*P* < 0.0001). However, due to the limitations of meta-analysis, which cannot control for all potential confounding factors, such as surgeon’s preference of surgical methods, cancer type, patient age, and overall health, further comprehensive and prospective comparative studies between the two methods on survival are still warranted.

Regarding the findings on reoperation and complication rates, although the meta-analysis showed no significant difference between the IF and P groups, some studies have indicated that the choice of specific surgical method can affect these outcomes in cases of metastatic bone disease. For instance, a recent meta-analysis by Putnam *et al.* on treatment modalities for pathologic fractures of the proximal femur revealed pooled reoperation rates of 9% for intramedullary nail (95% CI: 5–14%) and 7% for prothesis reconstruction (95% CI: 5–11%) across 16 studies involving 1,414 patients (55). Comparatively, our study utilized multiple databases, including PubMed, Embase, and Cochrane – expanding beyond Putnam *et al.*’s their PubMed-only search strategy – encompassing a broader range of articles, resulting in a more comprehensive evidence pool with lower heterogeneity (lower *I*^2^), indicating more consistent study selection. While we observed no significant differences in overall complication rates, our analysis comprehensively compared specific surgical methods, revealing procedure-specific complications in addition to the reported reoperation rates.

Iljazi *et al.* (56) further reported that DHS and cannulated screws were associated with higher revision rates than intramedullary nailing. Subtrochanteric lesions also showed a greater risk of mechanical failure. These findings support our subgroup analysis by illustrating how implant type and lesion location may influence surgical outcomes beyond overall complication rates.

The meta-analysis highlights several critical limitations in the current literature on surgical methods for proximal femoral metastases. Many studies were non-randomized and retrospective, limiting their evidential strength compared with randomized, prospective studies. Variability in follow-up durations affected the assessment of long-term outcomes, which cause high heterogeneity of the MSTS score, while inconsistent reporting of surgical techniques and outcomes hindered thorough comparative analysis. In addition, patient selection bias was evident, with differences in baseline health status influencing postoperative outcomes and reoperation rates. Despite these issues, our article provides a systematic analysis of the two surgical methods based on existing literature. Given the ongoing advancements in oncologic care, more comprehensive and prospective studies are needed to support decision-making for these surgical interventions.

## Conclusion

The study indicates that prosthesis reconstruction may offer superior long-term functional outcomes and extended survival compared with IF, while maintaining similar rates of complications and reoperations. However, survival differences across studies suggest that factors, such as selection bias – where patients with better expected survival and baseline physiology are more likely to receive prosthetic reconstruction – along with cancer type, patient age, and overall health status, can significantly influence survival outcomes. Selection bias is one of the most evident and important confounding factors in the literature on this topic, profoundly affecting the interpretation of findings. This highlights the need for more rigorous, prospective research to establish clearer guidelines for the treatment of proximal femoral metastases.

## Supplementary materials





















## ICMJE Statement of Interest

The authors declare that there is no conflict of interest that could be perceived as prejudicing the impartiality of the work reported.

## Funding Statement

Wan Fang Hospital provided financial support for this work (grant number 114-wf-eva-09 and w618).

## Data availability

All the information presented in this study can be found in the article. If you need additional details, please reach out to the corresponding author.
