# Supplementary material for: Comparative outcomes of internal fixation versus prosthetic reconstruction in the treatment of proximal femoral metastases: a systematic review and meta-analysis
Source: EFORT Open Rev. 2025 Nov 3;10(11):842–50. doi: 10.1530/EOR-2024-0131 (PMC12587033; doi:10.1530/EOR-2024-0131)
Supplement: Supplementary file 1 [file supplementary_figure_1.pdf]

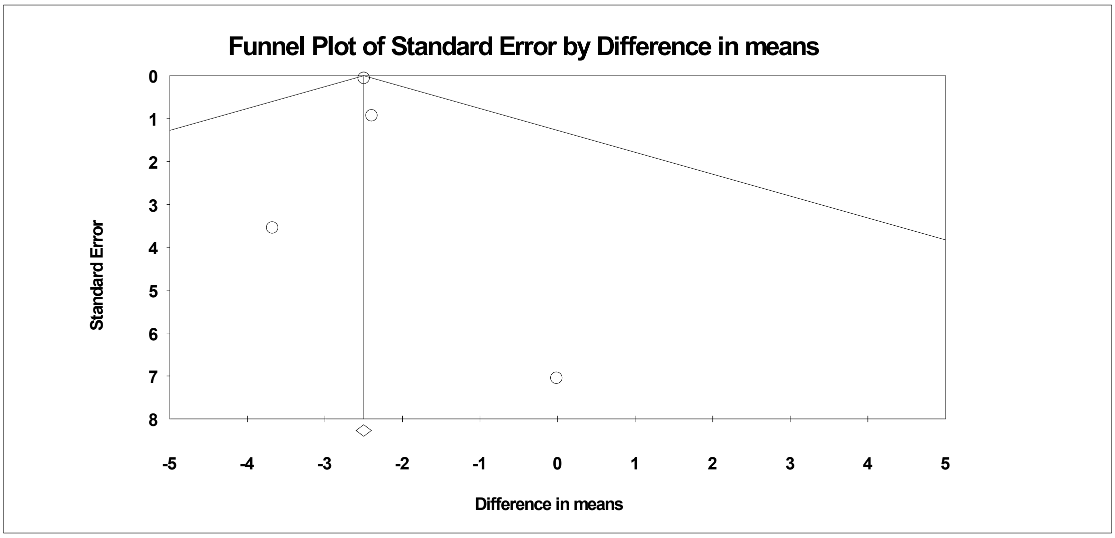

Supplementary Figure 1. Funnel plots of survival time[1-4]

The funnel plot demonstrates the absence of publication bias in the included studies analyzing survival time. The distribution of effect sizes is symmetrical, supported by Egger's test (p = 0.85).

Refence list of Supplementary Figure 1.

1. Yu, Z., Y. Xiong, R. Shi, L. Min, W. Zhang, H. Liu, X. Fang, C. Tu, and H. Duan, *Surgical management of metastatic lesions of the proximal femur with pathological fractures using intramedullary nailing or endoprosthetic replacement.* Mol Clin Oncol, 2018. **8**(1): p. 107-114.
2. Gao, H., Z. Liu, B. Wang, and A. Guo, *Clinical and functional comparison of endoprosthetic replacement with intramedullary nailing for treating proximal femur metastasis.* Chin J Cancer Res, 2016. **28**(2): p. 209-14.
3. Weiss, R.J., W. Ekström, B.H. Hansen, J. Keller, M. Laitinen, C. Trovik, O. Zaikova, and R. Wedin, *Pathological subtrochanteric fractures in 194 patients: a comparison of outcome after surgical treatment of pathological and non-pathological fractures.* J Surg Oncol, 2013. **107**(5): p. 498-504.
4. Zacherl, M., G. Gruber, M. Glehr, P. Ofner-Kopeinig, R. Radl, M. Greitbauer, V. Vecsei, and R. Windhager, *Surgery for pathological proximal femoral fractures, excluding femoral head and neck fractures: resection vs. stabilisation.* Int Orthop, 2011. **35**(10): p. 1537-43.
