# Supplementary material for: Comparative outcomes of internal fixation versus prosthetic reconstruction in the treatment of proximal femoral metastases: a systematic review and meta-analysis
Source: EFORT Open Rev. 2025 Nov 3;10(11):842–50. doi: 10.1530/EOR-2024-0131 (PMC12587033; doi:10.1530/EOR-2024-0131)
Supplement: Supplementary file 2 [file supplementary_figure_2.pdf]

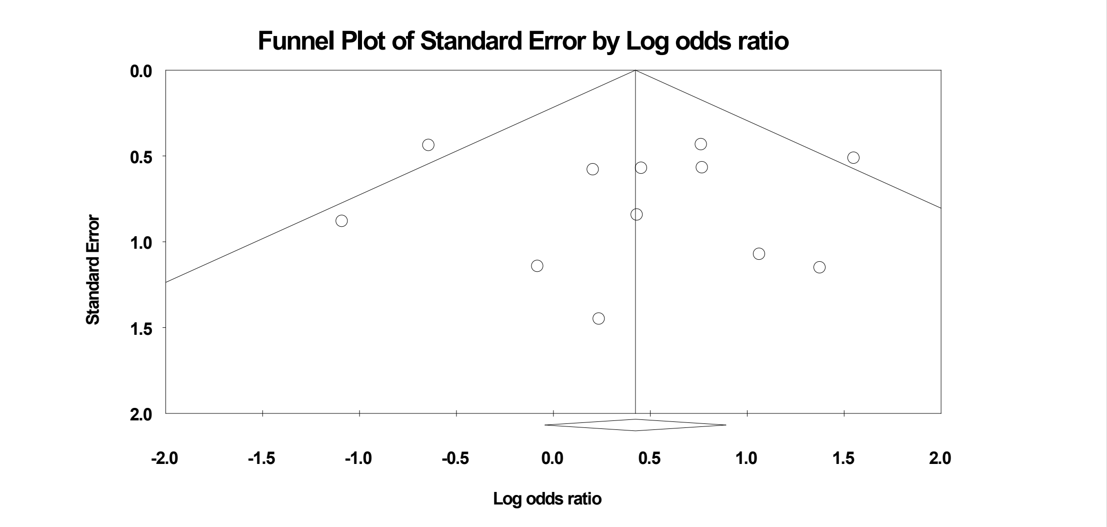


**Supplementary Figure 2. Funnel plots of reoperation rate[1-11]**

This funnel plot evaluates the potential publication bias for studies analyzing reoperation rates. The plot indicates a symmetrical distribution of study results, and Egger's test shows no significant bias ($p = 0.98$).

**Refence list of Supplementary Figure 2.**

1. Gusho, C.A., B. Clayton, N. Mehta, W. Hmeidan, M.W. Colman, S. Gitelis, and A.T. Blank, *Internal fixation versus endoprosthetic replacement of the proximal femur for metastatic bone disease: Single institutional outcomes.* J Orthop, 2021. **28**: p. 86-90.
2. Vitiello, R., C. Perisano, T. Greco, L. Cianni, C. Polichetti, R.M. Comodo, I. De Martino, V. La Vergata, and G. Maccauro, *Intramedullary nailing vs modular megaprosthesis in extracapsular metastases of proximal femur: clinical outcomes and complication in a retrospective study.* BMC Musculoskelet Disord, 2022. **22**(Suppl 2): p. 1069.
3. Tanaka, A., M. Okamoto, M. Kito, Y. Yoshimura, K. Aoki, S. Suzuki, A. Takazawa, and J. Takahashi, *Points of consideration when performing surgical procedures for proximal femoral bone metastasis.* J Orthop Sci, 2022. **27**(1): p. 229-234.
4. Sørensen, M.S., P.F. Horstmann, K. Hindsø, and M.M. Petersen, *Use of endoprostheses for proximal femur metastases results in a rapid rehabilitation and low risk of implant failure. A prospective population-based study.* J Bone Oncol, 2019. **19**: p. 100264.
5. Guzik, G., *Oncological and functional results after surgical treatment of bone metastases at the proximal femur.* BMC Surgery, 2018. **18**(1): p. 5.
6. Janssen, S.J., T. Teunis, F.J. Hornicek, C.N. van Dijk, J.A.M. Bramer, and J.H. Schwab, *Outcome after fixation of metastatic proximal femoral fractures: A*

*systematic review of 40 studies.* Journal of Surgical Oncology, 2016. **114**(4): p. 507-519.

7. Weiss, R.J., W. Ekström, B.H. Hansen, J. Keller, M. Laitinen, C. Trovik, O. Zaikova, and R. Wedin, *Pathological subtrochanteric fractures in 194 patients: a comparison of outcome after surgical treatment of pathological and non-pathological fractures.* J Surg Oncol, 2013. **107**(5): p. 498-504.
8. Harvey, N., E.R. Ahlmann, D.C. Allison, L. Wang, and L.R. Menendez, *Endoprostheses last longer than intramedullary devices in proximal femur metastases.* Clin Orthop Relat Res, 2012. **470**(3): p. 684-91.
9. Zacherl, M., G. Gruber, M. Glehr, P. Ofner-Kopeinig, R. Radl, M. Greitbauer, V. Vecsei, and R. Windhager, *Surgery for pathological proximal femoral fractures, excluding femoral head and neck fractures: resection vs. stabilisation.* Int Orthop, 2011. **35**(10): p. 1537-43.
10. Parker, M.J., A.Z. Khan, and T.K. Rowlands, *Survival after pathological fractures of the proximal femur.* Hip Int, 2011. **21**(5): p. 526-30.
11. Wedin, R. and H.C. Bauer, *Surgical treatment of skeletal metastatic lesions of the proximal femur: endoprosthesis or reconstruction nail?* J Bone Joint Surg Br, 2005. **87**(12): p. 1653-7.
