# Supplementary material for: Comparative outcomes of internal fixation versus prosthetic reconstruction in the treatment of proximal femoral metastases: a systematic review and meta-analysis
Source: EFORT Open Rev. 2025 Nov 3;10(11):842–50. doi: 10.1530/EOR-2024-0131 (PMC12587033; doi:10.1530/EOR-2024-0131)
Supplement: Supplementary file 3 [file supplementary_figure_3.pdf]

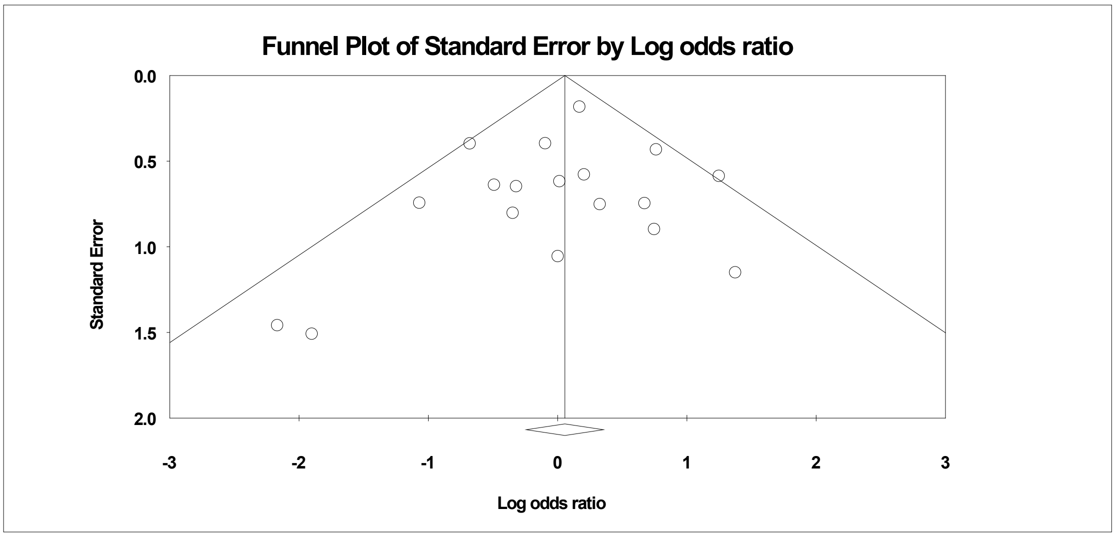

Supplementary Figure 3. Funnel plot of complication rates[1-18]

The funnel plot shows the absence of significant publication bias for complication rates. The symmetrical distribution of effect sizes is confirmed by Egger's test (p = 0.49).

Refence list of Supplementary Figure 3.

1. Gusho, C.A., B. Clayton, N. Mehta, W. Hmeidan, M.W. Colman, S. Gitelis, and A.T. Blank, *Internal fixation versus endoprosthetic replacement of the proximal femur for metastatic bone disease: Single institutional outcomes.* J Orthop, 2021. **28**: p. 86-90.
2. Terakawa, F., H. Kamoda, T. Yonemoto, Y. Hagiwara, T. Tsukanishi, H. Kinoshita, S. Ohtori, and T. Ishii, *Analysis of implants for metastatic bone tumors of the proximal femur: A retrospective study.* Asia Pac J Clin Oncol, 2023. **19**(5): p. e320-e325.
3. Vitiello, R., C. Perisano, T. Greco, L. Cianni, C. Polichetti, R.M. Comodo, I. De Martino, V. La Vergata, and G. Maccauro, *Intramedullary nailing vs modular megaprosthesis in extracapsular metastases of proximal femur: clinical outcomes and complication in a retrospective study.* BMC Musculoskelet Disord, 2022. **22**(Suppl 2): p. 1069.
4. Tanaka, A., M. Okamoto, M. Kito, Y. Yoshimura, K. Aoki, S. Suzuki, A. Takazawa, and J. Takahashi, *Points of consideration when performing surgical procedures for proximal femoral bone metastasis.* J Orthop Sci, 2022. **27**(1): p. 229-234.
5. Meynard, P., A. Seguineau, P. Laumonerie, T. Fabre, D. Foltran, L. Niglis, J. Descamps, C. Bouthors, M. Lebaron, C. Szymanski, F. Sailhan, and P. Bonnevialle, *Surgical management of proximal femoral metastasis: Fixation*

or hip replacement? A 309 case series. Orthop Traumatol Surg Res, 2020. **106**(6): p. 1013-1023.

6. Sørensen, M.S., P.F. Horstmann, K. Hindsø, and M.M. Petersen, *Use of endoprostheses for proximal femur metastases results in a rapid rehabilitation and low risk of implant failure. A prospective population-based study.* J Bone Oncol, 2019. **19**: p. 100264.
7. Yu, Z., Y. Xiong, R. Shi, L. Min, W. Zhang, H. Liu, X. Fang, C. Tu, and H. Duan, *Surgical management of metastatic lesions of the proximal femur with pathological fractures using intramedullary nailing or endoprosthetic replacement.* Mol Clin Oncol, 2018. **8**(1): p. 107-114.
8. Guzik, G., *Oncological and functional results after surgical treatment of bone metastases at the proximal femur.* BMC Surgery, 2018. **18**(1): p. 5.
9. Angelini, A., G. Trovarelli, A. Berizzi, E. Pala, A. Breda, M. Maraldi, and P. Ruggieri, *Treatment of pathologic fractures of the proximal femur.* Injury, 2018. **49 Suppl 3**: p. S77-s83.
10. Tsuda, Y., H. Yasunaga, H. Horiguchi, K. Fushimi, H. Kawano, and S. Tanaka, *Complications and Postoperative Mortality Rate After Surgery for Pathological Femur Fracture Related to Bone Metastasis: Analysis of a Nationwide Database.* Ann Surg Oncol, 2016. **23**(3): p. 801-10.
11. Janssen, S.J., T. Teunis, F.J. Hornicek, C.N. van Dijk, J.A.M. Bramer, and J.H. Schwab, *Outcome after fixation of metastatic proximal femoral fractures: A systematic review of 40 studies.* Journal of Surgical Oncology, 2016. **114**(4): p. 507-519.
12. Gao, H., Z. Liu, B. Wang, and A. Guo, *Clinical and functional comparison of endoprosthetic replacement with intramedullary nailing for treating proximal femur metastasis.* Chin J Cancer Res, 2016. **28**(2): p. 209-14.
13. Weiss, R.J., W. Ekström, B.H. Hansen, J. Keller, M. Laitinen, C. Trovik, O. Zaikova, and R. Wedin, *Pathological subtrochanteric fractures in 194 patients: a comparison of outcome after surgical treatment of pathological and non-pathological fractures.* J Surg Oncol, 2013. **107**(5): p. 498-504.
14. Fakler, J.K., F. Hase, J. Böhme, and C. Josten, *Safety aspects in surgical treatment of pathological fractures of the proximal femur - modular endoprosthetic replacement vs. intramedullary nailing.* Patient Saf Surg, 2013. **7**(1): p. 37.
15. Harvey, N., E.R. Ahlmann, D.C. Allison, L. Wang, and L.R. Menendez, *Endoprostheses last longer than intramedullary devices in proximal femur metastases.* Clin Orthop Relat Res, 2012. **470**(3): p. 684-91.

16. Zacherl, M., G. Gruber, M. Glehr, P. Ofner-Kopeinig, R. Radl, M. Greitbauer, V. Vecsei, and R. Windhager, *Surgery for pathological proximal femoral fractures, excluding femoral head and neck fractures: resection vs. stabilisation.* Int Orthop, 2011. **35**(10): p. 1537-43.
17. Parker, M.J., A.Z. Khan, and T.K. Rowlands, *Survival after pathological fractures of the proximal femur.* Hip Int, 2011. **21**(5): p. 526-30.
18. Wedin, R. and H.C. Bauer, *Surgical treatment of skeletal metastatic lesions of the proximal femur: endoprosthesis or reconstruction nail?* J Bone Joint Surg Br, 2005. **87**(12): p. 1653-7.
