# Supplementary material for: Comparative outcomes of internal fixation versus prosthetic reconstruction in the treatment of proximal femoral metastases: a systematic review and meta-analysis
Source: EFORT Open Rev. 2025 Nov 3;10(11):842–50. doi: 10.1530/EOR-2024-0131 (PMC12587033; doi:10.1530/EOR-2024-0131)
Supplement: Supplementary file 4 [file supplementary_figure_4.pdf]

Study name	Statistics for each study						
	Difference in means	Standard error	Variance	Lower limit	Upper limit	Z-Value	p-Value
2023 Terakawa	-49.28	14.46	209.01	-77.62	-20.95	-3.41	0.00
2022 Tanaka	-126.87	12.81	164.07	-151.97	-101.76	-9.90	0.00
2022 Vitiello	-54.00	12.82	164.25	-79.12	-28.88	-4.21	0.00
2021 Gusho	-63.52	8.85	78.33	-80.87	-46.18	-7.18	0.00
2019 S?rensen	-20.01	11.35	128.79	-42.25	2.23	-1.76	0.08
2018 Yu	-43.90	4.83	23.32	-53.36	-34.44	-9.09	0.00
2016 Gao	-15.90	2.40	5.74	-20.59	-11.21	-6.64	0.00
2016 Janssen	-47.76	6.59	43.47	-60.68	-34.84	-7.24	0.00
2013 Fakler	-51.52	28.20	795.44	-106.80	3.76	-1.83	0.07
	-51.49	9.95	98.92	-70.99	-32.00	-5.18	0.00

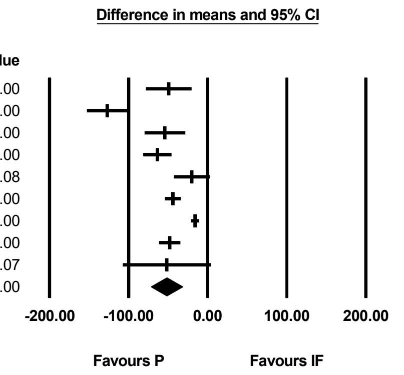

Meta Analysis

Supplementary Figure 4. Forest plot of operative time[1-9]

This forest plot compares operative times between internal fixation (IF) and prosthetic reconstruction (P). The results favor internal fixation (IF) with significantly shorter operative times. High heterogeneity ($I^2 = 93.76\%$) was observed, and Egger's test suggested publication bias ($p = 0.03$), which was adjusted using the trim-and-fill method.

Refence list of Supplementary Figure 4.

1. Terakawa, F., H. Kamoda, T. Yonemoto, Y. Hagiwara, T. Tsukanishi, H. Kinoshita, S. Ohtori, and T. Ishii, *Analysis of implants for metastatic bone tumors of the proximal femur: A retrospective study.* Asia Pac J Clin Oncol, 2023. **19**(5): p. e320-e325.
2. Vitiello, R., C. Perisano, T. Greco, L. Cianni, C. Polichetti, R.M. Comodo, I. De Martino, V. La Vergata, and G. Maccauro, *Intramedullary nailing vs modular megaprosthesis in extracapsular metastases of proximal femur: clinical outcomes and complication in a retrospective study.* BMC Musculoskelet Disord, 2022. **22**(Suppl 2): p. 1069.
3. Tanaka, A., M. Okamoto, M. Kito, Y. Yoshimura, K. Aoki, S. Suzuki, A. Takazawa, and J. Takahashi, *Points of consideration when performing surgical procedures for proximal femoral bone metastasis.* J Orthop Sci, 2022. **27**(1): p. 229-234.
4. Gusho, C.A., B. Clayton, N. Mehta, W. Hmeidan, M.W. Colman, S. Gitelis, and A.T. Blank, *Internal fixation versus endoprosthetic replacement of the proximal femur for metastatic bone disease: Single institutional outcomes.* J Orthop, 2021. **28**: p. 86-90.

5. Sørensen, M.S., P.F. Horstmann, K. Hindsø, and M.M. Petersen, *Use of endoprostheses for proximal femur metastases results in a rapid rehabilitation and low risk of implant failure. A prospective population-based study.* J Bone Oncol, 2019. **19**: p. 100264.
6. Yu, Z., Y. Xiong, R. Shi, L. Min, W. Zhang, H. Liu, X. Fang, C. Tu, and H. Duan, *Surgical management of metastatic lesions of the proximal femur with pathological fractures using intramedullary nailing or endoprosthetic replacement.* Mol Clin Oncol, 2018. **8**(1): p. 107-114.
7. Janssen, S.J., T. Teunis, F.J. Hornicek, C.N. van Dijk, J.A.M. Bramer, and J.H. Schwab, *Outcome after fixation of metastatic proximal femoral fractures: A systematic review of 40 studies.* Journal of Surgical Oncology, 2016. **114**(4): p. 507-519.
8. Gao, H., Z. Liu, B. Wang, and A. Guo, *Clinical and functional comparison of endoprosthetic replacement with intramedullary nailing for treating proximal femur metastasis.* Chin J Cancer Res, 2016. **28**(2): p. 209-14.
9. Fakler, J.K., F. Hase, J. Böhme, and C. Josten, *Safety aspects in surgical treatment of pathological fractures of the proximal femur - modular endoprosthetic replacement vs. intramedullary nailing.* Patient Saf Surg, 2013. **7**(1): p. 37.
