# Supplementary material for: Comparative outcomes of internal fixation versus prosthetic reconstruction in the treatment of proximal femoral metastases: a systematic review and meta-analysis
Source: EFORT Open Rev. 2025 Nov 3;10(11):842–50. doi: 10.1530/EOR-2024-0131 (PMC12587033; doi:10.1530/EOR-2024-0131)
Supplement: Supplementary file 5 [file supplementary_figure_5.pdf]

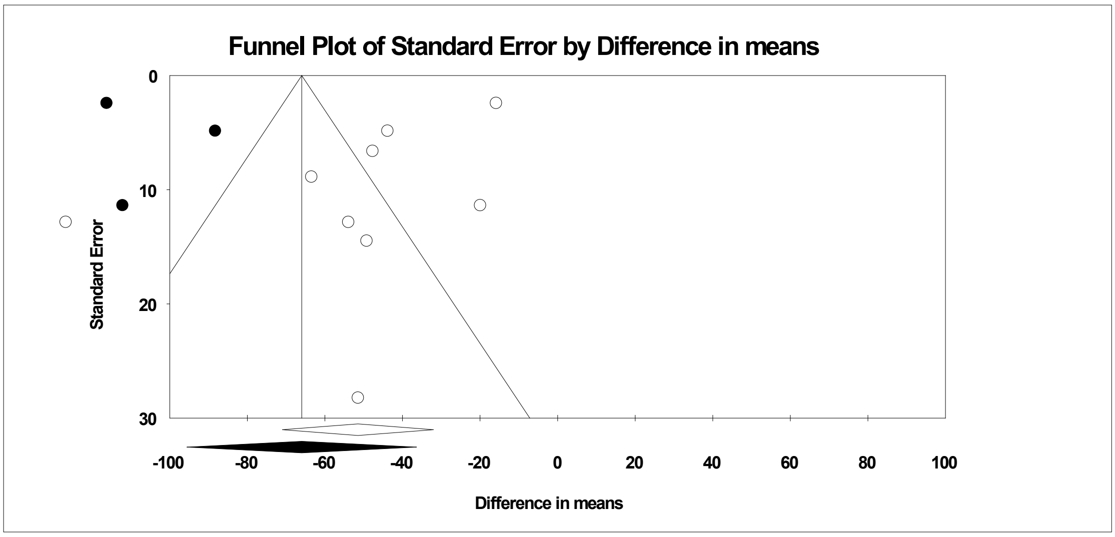

Supplementary Figure 5. Funnel plot of operative time with trim-and-fill adjustment[1-9]

This funnel plot illustrates the results after applying the trim-and-fill method to account for potential publication bias in operative time comparisons. The adjusted plot shows reduced asymmetry, improving the reliability of the findings.

Refence list of Supplementary Figure 5.

1. Terakawa, F., H. Kamoda, T. Yonemoto, Y. Hagiwara, T. Tsukanishi, H. Kinoshita, S. Ohtori, and T. Ishii, *Analysis of implants for metastatic bone tumors of the proximal femur: A retrospective study.* Asia Pac J Clin Oncol, 2023. **19**(5): p. e320-e325.
2. Vitiello, R., C. Perisano, T. Greco, L. Cianni, C. Polichetti, R.M. Comodo, I. De Martino, V. La Vergata, and G. Maccauro, *Intramedullary nailing vs modular megaprosthesis in extracapsular metastases of proximal femur: clinical outcomes and complication in a retrospective study.* BMC Musculoskelet Disord, 2022. **22**(Suppl 2): p. 1069.
3. Tanaka, A., M. Okamoto, M. Kito, Y. Yoshimura, K. Aoki, S. Suzuki, A. Takazawa, and J. Takahashi, *Points of consideration when performing surgical procedures for proximal femoral bone metastasis.* J Orthop Sci, 2022. **27**(1): p. 229-234.
4. Gusho, C.A., B. Clayton, N. Mehta, W. Hmeidan, M.W. Colman, S. Gitelis, and A.T. Blank, *Internal fixation versus endoprosthetic replacement of the proximal femur for metastatic bone disease: Single institutional outcomes.* J Orthop, 2021. **28**: p. 86-90.
5. Sørensen, M.S., P.F. Horstmann, K. Hindsø, and M.M. Petersen, *Use of endoprostheses for proximal femur metastases results in a rapid*

rehabilitation and low risk of implant failure. A prospective population-based study. J Bone Oncol, 2019. **19**: p. 100264.

6. Yu, Z., Y. Xiong, R. Shi, L. Min, W. Zhang, H. Liu, X. Fang, C. Tu, and H. Duan, *Surgical management of metastatic lesions of the proximal femur with pathological fractures using intramedullary nailing or endoprosthetic replacement.* Mol Clin Oncol, 2018. **8**(1): p. 107-114.
7. Janssen, S.J., T. Teunis, F.J. Hornicek, C.N. van Dijk, J.A.M. Bramer, and J.H. Schwab, *Outcome after fixation of metastatic proximal femoral fractures: A systematic review of 40 studies.* Journal of Surgical Oncology, 2016. **114**(4): p. 507-519.
8. Gao, H., Z. Liu, B. Wang, and A. Guo, *Clinical and functional comparison of endoprosthetic replacement with intramedullary nailing for treating proximal femur metastasis.* Chin J Cancer Res, 2016. **28**(2): p. 209-14.
9. Fakler, J.K., F. Hase, J. Böhme, and C. Josten, *Safety aspects in surgical treatment of pathological fractures of the proximal femur - modular endoprosthetic replacement vs. intramedullary nailing.* Patient Saf Surg, 2013. **7**(1): p. 37.
