# Supplementary material for: Comparative outcomes of internal fixation versus prosthetic reconstruction in the treatment of proximal femoral metastases: a systematic review and meta-analysis
Source: EFORT Open Rev. 2025 Nov 3;10(11):842–50. doi: 10.1530/EOR-2024-0131 (PMC12587033; doi:10.1530/EOR-2024-0131)
Supplement: Supplementary file 6 [file supplementary_figure_6.pdf]

Study name	Difference in means	Standard error	Variance	Lower limit	Upper limit	Z-Value	p-Value
2023 Terakawa	-210.88	94.24	8881.03	-395.59	-26.18	-2.24	0.03
2022 Tanaka	-554.12	102.46	10498.46	-754.94	-353.29	-5.41	0.00
2021 Gusho	-386.03	47.87	2291.64	-479.85	-292.20	-8.06	0.00
2019 S?rensen	-199.85	63.80	4070.94	-324.91	-74.80	-3.13	0.00
2018 Yu	-286.30	20.61	424.66	-326.69	-245.91	-13.89	0.00
2016 Gao	-182.40	41.16	1693.94	-263.07	-101.73	-4.43	0.00
2016 Janssen	-225.80	32.12	1031.84	-288.76	-162.84	-7.03	0.00
	-275.33	33.35	1112.16	-340.69	-209.96	-8.26	0.00

Difference in means and 95% CI

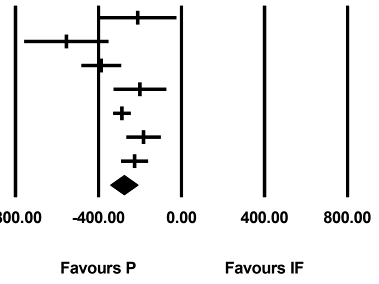

Meta Analysis

Supplementary Figure 6. Forest plot of blood loss [1-7]

The forest plot compares blood loss during internal fixation (IF) versus prosthetic reconstruction (P). Internal fixation (IF) demonstrated significantly lower blood loss ($p < 0.001$). Moderate heterogeneity was observed ($I^2 = 72.99\%$), and no significant publication bias was detected (Egger's test $p = 0.77$).

Refence list of Supplementary Figure 6.

1. Terakawa, F., H. Kamoda, T. Yonemoto, Y. Hagiwara, T. Tsukanishi, H. Kinoshita, S. Ohtori, and T. Ishii, *Analysis of implants for metastatic bone tumors of the proximal femur: A retrospective study.* Asia Pac J Clin Oncol, 2023. **19**(5): p. e320-e325.
2. Tanaka, A., M. Okamoto, M. Kito, Y. Yoshimura, K. Aoki, S. Suzuki, A. Takazawa, and J. Takahashi, *Points of consideration when performing surgical procedures for proximal femoral bone metastasis.* J Orthop Sci, 2022. **27**(1): p. 229-234.
3. Gusho, C.A., B. Clayton, N. Mehta, W. Hmeidan, M.W. Colman, S. Gitelis, and A.T. Blank, *Internal fixation versus endoprosthetic replacement of the proximal femur for metastatic bone disease: Single institutional outcomes.* J Orthop, 2021. **28**: p. 86-90.
4. Sørensen, M.S., P.F. Horstmann, K. Hindsø, and M.M. Petersen, *Use of endoprostheses for proximal femur metastases results in a rapid rehabilitation and low risk of implant failure. A prospective population-based study.* J Bone Oncol, 2019. **19**: p. 100264.
5. Yu, Z., Y. Xiong, R. Shi, L. Min, W. Zhang, H. Liu, X. Fang, C. Tu, and H. Duan, *Surgical management of metastatic lesions of the proximal femur with pathological fractures using intramedullary nailing or endoprosthetic replacement.* Mol Clin Oncol, 2018. **8**(1): p. 107-114.

6. Janssen, S.J., T. Teunis, F.J. Hornicek, C.N. van Dijk, J.A.M. Bramer, and J.H. Schwab, *Outcome after fixation of metastatic proximal femoral fractures: A systematic review of 40 studies.* Journal of Surgical Oncology, 2016. **114**(4): p. 507-519.
7. Gao, H., Z. Liu, B. Wang, and A. Guo, *Clinical and functional comparison of endoprosthetic replacement with intramedullary nailing for treating proximal femur metastasis.* Chin J Cancer Res, 2016. **28**(2): p. 209-14.
