# Supplementary material for: Comparative outcomes of internal fixation versus prosthetic reconstruction in the treatment of proximal femoral metastases: a systematic review and meta-analysis
Source: EFORT Open Rev. 2025 Nov 3;10(11):842–50. doi: 10.1530/EOR-2024-0131 (PMC12587033; doi:10.1530/EOR-2024-0131)
Supplement: Supplementary file 7 [file supplementary_figure_7.pdf]

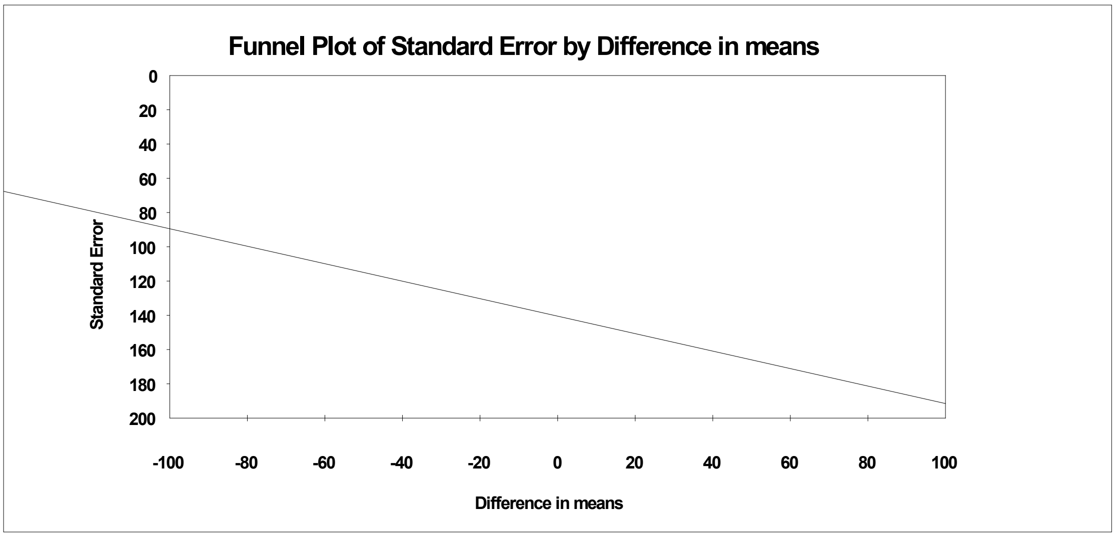


**Supplementary Figure 7. Funnel plot of blood loss[1-7]**

This funnel plot assesses publication bias for blood loss comparisons. The symmetrical distribution of effect sizes supports the absence of significant bias, as confirmed by Egger's test (p = 0.77).

**Refence list of Supplementary Figure 7.**


1. Terakawa, F., H. Kamoda, T. Yonemoto, Y. Hagiwara, T. Tsukanishi, H. Kinoshita, S. Ohtori, and T. Ishii, *Analysis of implants for metastatic bone tumors of the proximal femur: A retrospective study.* Asia Pac J Clin Oncol, 2023. **19**(5): p. e320-e325.
2. Tanaka, A., M. Okamoto, M. Kito, Y. Yoshimura, K. Aoki, S. Suzuki, A. Takazawa, and J. Takahashi, *Points of consideration when performing surgical procedures for proximal femoral bone metastasis.* J Orthop Sci, 2022. **27**(1): p. 229-234.
3. Gusho, C.A., B. Clayton, N. Mehta, W. Hmeidan, M.W. Colman, S. Gitelis, and A.T. Blank, *Internal fixation versus endoprosthetic replacement of the proximal femur for metastatic bone disease: Single institutional outcomes.* J Orthop, 2021. **28**: p. 86-90.
4. Sørensen, M.S., P.F. Horstmann, K. Hindsø, and M.M. Petersen, *Use of endoprostheses for proximal femur metastases results in a rapid rehabilitation and low risk of implant failure. A prospective population-based study.* J Bone Oncol, 2019. **19**: p. 100264.
5. Yu, Z., Y. Xiong, R. Shi, L. Min, W. Zhang, H. Liu, X. Fang, C. Tu, and H. Duan, *Surgical management of metastatic lesions of the proximal femur with pathological fractures using intramedullary nailing or endoprosthetic replacement.* Mol Clin Oncol, 2018. **8**(1): p. 107-114.

6. Janssen, S.J., T. Teunis, F.J. Hornicek, C.N. van Dijk, J.A.M. Bramer, and J.H. Schwab, *Outcome after fixation of metastatic proximal femoral fractures: A systematic review of 40 studies.* Journal of Surgical Oncology, 2016. **114**(4): p. 507-519.
7. Gao, H., Z. Liu, B. Wang, and A. Guo, *Clinical and functional comparison of endoprosthetic replacement with intramedullary nailing for treating proximal femur metastasis.* Chin J Cancer Res, 2016. **28**(2): p. 209-14.
