# Supplementary material for: Comparative outcomes of internal fixation versus prosthetic reconstruction in the treatment of proximal femoral metastases: a systematic review and meta-analysis
Source: EFORT Open Rev. 2025 Nov 3;10(11):842–50. doi: 10.1530/EOR-2024-0131 (PMC12587033; doi:10.1530/EOR-2024-0131)
Supplement: Supplementary file 8 [file supplementary_materials.pdf]

Supplementary Material: Search Strategy

This file provides a detailed description of the literature search strategies used in our systematic review and meta-analysis on internal fixation versus prosthetic reconstruction for proximal femoral metastases. Searches were conducted in PubMed, Embase, and the Cochrane Library. The last search was conducted on December 31, 2023. No language or date restrictions were applied during the initial database search. Only English-language and comparative original journal articles were included in the final analysis.

PubMed (n = 354)

Search Strategy:

(("randomized controlled trial"[pt] OR "controlled clinical trial"[pt] OR "clinical trials as topic"[mesh] OR "random allocation"[mesh] OR "double-blind method"[mesh] OR "single-blind method"[mesh] OR "clinical trial"[pt] OR "research design"[mesh:noexp] OR "comparative study"[pt] OR "evaluation studies"[pt] OR "follow-up studies"[mesh] OR "prospective studies"[mesh] OR "cross-over studies"[mesh] OR "clinical trial"[tw] OR ((singl*[tw] OR doubl*[tw] OR trebl*[tw]) AND (mask*[tw] OR blind*[tw])) OR placebo*[tw] OR random*[tw] OR "control"[tw] OR "controls"[tw] OR prospectiv*[tw] OR volunteer*[tw]) OR ("cohort studies"[mesh] OR "case-control studies"[mesh] OR "comparative study"[pt] OR "risk factors"[mesh] OR "cohort"[tw] OR "compared"[tw] OR "groups"[tw] OR "case control"[tw] OR "multivariate"[tw])) AND (proximal) AND ((femur) OR (femoral)) AND ((metastasis) OR (metastases))

Embase (n = 857)

Search Strategy:

('clinical article'/exp OR 'controlled study'/exp OR 'major clinical study'/exp OR 'prospective study'/exp OR 'cohort analysis'/exp OR 'cohort':ti,ab OR 'compared':ti,ab OR 'groups':ti,ab OR 'case control':ti,ab OR 'multivariate':ti,ab OR ('clinical':ti,ab AND 'trial':ti,ab) OR 'clinical trial'/exp OR 'controlled clinical trial'/exp OR randomized:ti,ab OR placebo:ti,ab OR 'drug therapy':lnk OR randomly:ti,ab OR trial:ti,ab OR groups:ti,ab OR random* OR 'randomized controlled trial'/exp) AND proximal AND (femur OR femoral) AND (metastasis OR metastases)

Cochrane Library (n = 20)

Search Strategy:

Searched using keyword combinations similar to those used in PubMed and Embase.

Search Summary

- Total records retrieved: 1231
- After duplicates removed: 946 (via EndNote and manual check)
- Articles excluded after title and abstract screening: [irrelevant, non-English, not original article, non-human, not comparative]
- Articles reviewed in full text: 51
- Articles included in final meta-analysis: 19
- Additional study included by citation searching: Weiss 2013

Note:
EndNote X9 was used to manage references and remove duplicates.
