# Supplementary material for: Comparative outcomes of internal fixation versus prosthetic reconstruction in the treatment of proximal femoral metastases: a systematic review and meta-analysis
Source: EFORT Open Rev. 2025 Nov 3;10(11):842–50. doi: 10.1530/EOR-2024-0131 (PMC12587033; doi:10.1530/EOR-2024-0131)
Supplement: Supplementary file 9 [file supplementary_table_1.pdf]

Year	Author	Country	Study design	Study period	Target	Follow-up time	Group			Characteristics	
							Group	Sample size	Definition	Age	Sex (M/F)
2023	Terakawa[1]	Japan	retrospective cohort	2007-2020	metastatic bone tumors of the proximal femur	580 ± 777.2	Internal fixation	41	Intramedullary nail (41)	67.6 ± 12.3	16/25
							Prosthesis	41	Bipolar hip arthroplasty (BHP) (32) Modular megaprosthesis (MMP) (9)	BHP: 66.5 ± 11.2 MMP: 65.4 ± 14.2	BHP: 15/17 MMP: 5/4
2022	Tanaka[2]	Japan	retrospective cohort	2009-2019	proximal femoral bone metastases	median: 14months (IQR 5-35)	Internal fixation	24	Intramedullary nails (23) Ender nails (1)	68 (58-72)	10/14
							Prosthesis	16	cemented modular endoprosthesis (13) hemiarthroplasty with bipolar head (2) total hip arthroplasty (1)	67 (60-72)	8/8
2022	Vitiello[3]	Italy	retrospective cohort	Jan 2016 - Dec 2020	Trochanteric metastases of the proximal femur	21.2 ± 16.3 months	Internal fixation	20	Intramedullary nailing (20)	69.4 ± 10	6/14
							Prosthesis	25	Hip megaprostheses implantation (25)	67.5 ± 9.9	10/15
2021	Gusho[4]	USA	retrospective cohort	2005-2019	Metastatic bone disease of the proximal femur	median: 8 months (IQR 0-60)	Internal fixation	94	Internal fixation	median: 61 (IQR 69-55)	40/54
							Prosthesis	19	Proximal femur replacement	median: 62 (IQR 74.5-54)	6/13
2020	Meynard[5]	France	retrospective cohort	Jan 2001 - Dec 2017	PFM in the cervicocephalic and trochanteric or subtrochanteric segments on the AO classification, to the exclusion of diaphyseal locations	Beyond 6 months	Internal fixation	148	Screwed plates (12) Intramedullary nails (136)	M: 67.2 ± 11.5 F: 62.5 ± 13.2	117/182
							Prosthesis	161	Partial hip prosthesis (22) Femoral implant-Conventional stem (100) Femoral implant-Long stem (11) Femoral implant-Modular stem (28) Acetabular implant-Dual mobility (83) Acetabular implant-Metal ring + dual mobility (38) Acetabular implant-Reinforced cup (27) Acetabular implant-Conventional (13)		
2019	Sørensen[6]	Denmark	prospective cohort	2014/5/19 - 2016/5/18	Metastatic lesions located in the proximal and/metaphysial part of the femur	768-1468 days	Internal fixation	44	cannulated-screws (1) plates (dynamic hip screw) (5) intramedullary nails (38)	median: 69 (IQR: 62 — 75)	21/23
							Prosthesis	66	conventional hip prostheses (38) hemiarthroplaty (18) total joint replacement (18) total joint replacement+partial pelvis replacement (2) tumour prostheses (26) intercalary spacers (2)	median: 66 (IQR: 58 — 77)	34/32
2018	Angelini[7]	Italy	prospective cohort	Oct 2015 - Dec 2017	Proximal femoral metastases treated for impending or actual pathological	10.2 months (range 6-26.3)	Internal fixation	7	Intramedullary nailing (7)	63.6 (range 35-92)	11/29
							Prosthesis	33	Endoprosthetic replacement (4) Proximal femur resection (29)		
2018	Guzik[8]	Poland	retrospective cohort	2010 - 2016	Metastatic tumour to the proximal femur	27 months (min 4, max 51)	Internal fixation	26	Intramedullary gamma nail (20) Dynamic Hip Screw (6)	M: 72 F: 67	45/77
							Prosthesis	96	Wide tumour resection and modular endoprosthetic replacement (75) Standard or long stem hip endoprosthetic replacement (21)		
2018	Yu[9]	China	retrospective cohort	Jan 2005 - Dec	pathological proximal femur	median: 12.9 months (range	Internal fixation	31	intramedullary nailing	60.2±6.6	19/12

					fractures	3-98)	Prosthesis	57	endoprosthetic	62.5±6.2	34/23
2016	Gao[10]	China	retrospective cohort	Jan 2007 - Dec 2014	Proximal femoral metastases	12.1± (range: 10-47 months)	Internal fixation	21	Intramedullary nailing (21)	72.6	20/14
							Prosthesis	13	Modular endoprosthetic replacement (13)	62.2	
2016	Janssen[11]	USA	retrospective cohort	Jan 1999 - Jan 2014	Proximal femoral metastasis or multiple myeloma	median: 4 months (range: 0-144)	Internal fixation	347	Intramedullary nailing (302)<br>Open reduction and internal fixation (45)	IMN-median: 62 (IQR: 52-70)<br>ORIF-median: 65 (IQR: 54-75)	IMN: 114/188<br>ORIF: 17/28
							Prosthesis	70	Endoprosthetic reconstruction (70)	median: 63 (IQR: 55-72)	32/38
2016	Tsuda[12]	Japan	retrospective cohort	2007-2012	pathological femur fracture related to bone metastasis		Internal fixation	1073	intramedullary nailing (769)<br>compression hip screw (53)<br>cannulated cancellous hip screw (38)<br>plate fixation (31)<br>data missing (182)	≦59: 179<br>60 — 69: 240<br>70 — 79: 309<br>≧80 455: 345	507/566
							Prosthesis	424	endoprosthetic reconstruction	≦59: 69<br>60 — 69: 89<br>70 — 79: 156<br>≧80 455: 110	169/255
2013	Fakler[13]	Germany	retrospective cohort	Jan 2003 - Dec 2012	Pathologic fractures due to bone metastasis in the intertrochanteric	median: 3.0 months (IQR 1.0-18.3)	Internal fixation	12	cephalomedullary nail (12)	median: 73.8 (IQR: 66.5-80.4)	
							Prosthesis	8	en-bloc resection and modular proximal femur replacement (8)	median: 61.9 (IQR: 59.5-72.7)	
2013	Weiss[14]	Sweden	prospective cohort	1999 - 2009	Pathological subtrochanteric femur fractures	medain: 6 months (0 – 119)	Internal fixation	108	Intramedullary nail (108)	median: 68 (29-96)	
							Prosthesis	82	Hemiarthroplasty (35)<br>Tumorprosthesis (24)<br>Total hip arthroplasty (23)		
2012	Harvey[15]	USA	retrospective cohort	1998 - 2009	Metastatic disease of the proximal femur	16 months (range: 0.25-86)	Internal fixation	46	Intramedullary nailing (46)	61 (range: 17-86)	19/26
							Prosthesis	113	Resection of the proximal femur with placement of an endoprosthesis (113)	56 (range: 16-91)	53/60
2012	Steensma[16]	USA	retrospective cohort	1993 - 2008	Impending or displaced pathologic femur fractures, excluding	NR	Internal fixation	101	Intramedullary nailing (82)<br>Open reduction-internal fixation (19)	IMN median: 61.8<br>ORIF median: 55.7	
							Prosthesis	197	Endoprosthetic reconstruction (197)	median: 62.4	
2011	Parker[17]	UK	retrospective cohort	Jan 1989 - Jul 2008	Metastatic proximal femoral fracture		Internal fixation	90	Multiple cancellous screws (15)<br>Sliding hip screw (35)<br>Short Intramedullary nail (8)<br>Long intramedullary nail (32)	72.2 (range: 4 -96)	57/90
							Prosthesis	54	Uncemented Moore hemiarthroplasty (25)<br>Cemented long stem bipolar hemiarthroplasty (12)<br>Cemented unipolar hemiarthroplasty (11)<br>Cemented total hip replacement (6)		
2011	Zacherl[18]	Austria	retrospective cohort	Nov 1998 - Mar 2004	Pathological proximal femoral fractures	mean: 8 months	Internal fixation	33	Intramedullary nail (33)	62	14/15
							Prosthesis	31	Resection and reconstruction (31)	65	10/20
2005	Wedin[19]	Sweden	retrospective cohort	1996-2003	Metastatic lesions of the proximal femur	median: 1.6 years	Internal fixation	37	Reconstruction nail (22)<br>DHS (12)<br>Locked intramedullary nail (2)<br>Hip screws (1)	median: 69 years (33 to 91)	69/73
							Prosthesis	109	Hemiarthroplasty (51)<br>Bipolar prosthesis (4)<br>Regular THR (45)<br>Tumour prosthesis (5)<br>Reconstruction prosthesis (4)		

References related to Supplementary Table 1

1. Terakawa, F., H. Kamoda, T. Yonemoto, Y. Hagiwara, T. Tsukanishi, H. Kinoshita, S. Ohtori, and T. Ishii, *Analysis of implants for metastatic bone tumors of the proximal femur: A retrospective study.* Asia Pac J Clin Oncol, 2023. **19**(5): p. e320-e325.
2. Tanaka, A., M. Okamoto, M. Kito, Y. Yoshimura, K. Aoki, S. Suzuki, A. Takazawa, and J. Takahashi, *Points of consideration when performing surgical procedures for proximal femoral bone metastasis.* J Orthop Sci, 2022. **27**(1): p. 229-234.
3. Vitiello, R., C. Perisano, T. Greco, L. Cianni, C. Polichetti, R.M. Comodo, I. De Martino, V. La Vergata, and G. Maccauro, *Intramedullary nailing vs modular megaprosthesis in extracapsular metastases of proximal femur: clinical outcomes and complication in a retrospective study.* BMC Musculoskelet Disord, 2022. **22**(Suppl 2): p. 1069.
4. Gusho, C.A., B. Clayton, N. Mehta, W. Hmeidan, M.W. Colman, S. Gitelis, and A.T. Blank, *Internal fixation versus endoprosthetic replacement of the proximal femur for metastatic bone disease: Single institutional outcomes.* J Orthop, 2021. **28**: p. 86-90.
5. Meynard, P., A. Seguineau, P. Laumonerie, T. Fabre, D. Foltran, L. Niglis, J. Descamps, C. Bouthors, M. Lebaron, C. Szymanski, F. Sailhan, and P. Bonnevialle, *Surgical management of proximal femoral metastasis: Fixation or hip replacement? A 309 case series.* Orthop Traumatol Surg Res, 2020. **106**(6): p. 1013-1023.
6. Sørensen, M.S., P.F. Horstmann, K. Hindsø, and M.M. Petersen, *Use of endoprostheses for proximal femur metastases results in a rapid rehabilitation and low risk of implant failure. A prospective population-based study.* J Bone Oncol, 2019. **19**: p. 100264.
7. Angelini, A., G. Trovarelli, A. Berizzi, E. Pala, A. Breda, M. Maraldi, and P. Ruggieri, *Treatment of pathologic fractures of the proximal femur.* Injury, 2018. **49 Suppl 3**: p. S77-s83.
8. Guzik, G., *Oncological and functional results after surgical treatment of bone metastases at the proximal femur.* BMC Surgery, 2018. **18**(1): p. 5.
9. Yu, Z., Y. Xiong, R. Shi, L. Min, W. Zhang, H. Liu, X. Fang, C. Tu, and H. Duan, *Surgical management of metastatic lesions of the proximal femur with pathological fractures using intramedullary nailing or endoprosthetic replacement.* Mol Clin Oncol, 2018. **8**(1): p. 107-114.
10. Gao, H., Z. Liu, B. Wang, and A. Guo, *Clinical and functional comparison of endoprosthetic replacement with intramedullary nailing for treating proximal femur metastasis.* Chin J Cancer Res, 2016. **28**(2): p. 209-14.
11. Janssen, S.J., T. Teunis, F.J. Hornicek, C.N. van Dijk, J.A.M. Bramer, and J.H. Schwab, *Outcome after fixation of metastatic proximal femoral fractures: A systematic review of 40 studies.* Journal of Surgical Oncology, 2016. **114**(4): p. 507-519.
12. Tsuda, Y., H. Yasunaga, H. Horiguchi, K. Fushimi, H. Kawano, and S. Tanaka, *Complications and Postoperative Mortality Rate After Surgery for Pathological Femur Fracture Related to Bone Metastasis: Analysis of a Nationwide Database.* Ann Surg Oncol, 2016. **23**(3): p. 801-10.
13. Fakler, J.K., F. Hase, J. Böhme, and C. Josten, *Safety aspects in surgical treatment of pathological fractures of the proximal femur - modular endoprosthetic replacement vs. intramedullary nailing.* Patient Saf Surg, 2013. **7**(1): p. 37.
14. Weiss, R.J., W. Ekström, B.H. Hansen, J. Keller, M. Laitinen, C. Trovik, O. Zaikova, and R. Wedin, *Pathological subtrochanteric fractures in 194 patients: a comparison of outcome after surgical treatment of pathological and non-pathological fractures.* J Surg Oncol, 2013. **107**(5): p. 498-504.
15. Harvey, N., E.R. Ahlmann, D.C. Allison, L. Wang, and L.R. Menendez, *Endoprostheses last longer than intramedullary devices in proximal femur metastases.* Clin Orthop Relat Res, 2012. **470**(3): p. 684-91.
16. Steensma, M., P.J. Boland, C.D. Morris, E. Athanasian, and J.H. Healey, *Endoprosthetic treatment is more durable for pathologic proximal femur fractures.* Clin Orthop Relat Res, 2012. **470**(3): p. 920-6.
17. Parker, M.J., A.Z. Khan, and T.K. Rowlands, *Survival after pathological fractures of the proximal femur.* Hip Int, 2011. **21**(5): p. 526-30.
18. Zacherl, M., G. Gruber, M. Glehr, P. Ofner-Kopeinig, R. Radl, M. Greitbauer, V. Vecsei, and R. Windhager, *Surgery for pathological proximal femoral fractures, excluding femoral head and neck fractures: resection vs. stabilisation.* Int Orthop, 2011. **35**(10): p. 1537-43.
19. Wedin, R. and H.C. Bauer, *Surgical treatment of skeletal metastatic lesions of the proximal femur: endoprosthesis or reconstruction nail?* J Bone Joint Surg Br, 2005. **87**(12): p. 1653-7.
