# Supplementary material for: Comparative outcomes of internal fixation versus prosthetic reconstruction in the treatment of proximal femoral metastases: a systematic review and meta-analysis
Source: EFORT Open Rev. 2025 Nov 3;10(11):842–50. doi: 10.1530/EOR-2024-0131 (PMC12587033; doi:10.1530/EOR-2024-0131)
Supplement: Supplementary file 10 [file supplementary_table_2.pdf]

## Supplementary Table – Newcastle–Ottawa Scale (NOS) Quality Assessment

| Study | Selection (max 4) | Comparability (max 2) | Outcome/Exposure (max 3) | Total Score (max 9) |
|---|---|---|---|---|
| Terakawa et al. (2023)[1] | 3 | 1 | 2 | 6 |
| Tanaka et al. (2022)[2] | 3 | 1 | 2 | 6 |
| Vitiello et al. (2022)[3] | 4 | 1 | 2 | 7 |
| Gusho et al. (2021)[4] | 3 | 1 | 2 | 6 |
| Meynard et al. (2020)[5] | 3 | 1 | 3 | 7 |
| Sørensen et al. (2019)[6] | 3 | 1 | 3 | 7 |
| Angelini et al. (2018)[7] | 3 | 1 | 2 | 6 |
| Guzik et al. (2018)[8] | 3 | 1 | 3 | 7 |
| Yu et al. (2018)[9] | 3 | 0 | 3 | 6 |
| Gao et al. (2016)[10] | 3 | 0 | 4 | 7 |
| Janssen et al. (2016) [11] | 4 | 1 | 3 | 8 |
| Tsuda et al. (2016)[12] | 4 | 2 | 3 | 9 |
| Fakler et al. (2013)[13] | 4 | 2 | 2 | 8 |
| Weiss et al. (2013)[14] | 4 | 2 | 2 | 8 |
| Harvey et al. (2012)[15] | 3 | 0 | 3 | 6 |
| Steensma et al. (2012)[16] | 4 | 1 | 2 | 7 |
| Parker et al. (2011)[17] | 4 | 1 | 2 | 7 |
| Zacherl et al. (2011)[18] | 4 | 1 | 2 | 7 |
| Wedin et al. (2005)[19] | 3 | 2 | 3 | 8 |

Note: The NOS includes three domains: selection (0–4 points), comparability (0–2 points), and outcome/exposure (0–3 points). A higher score indicates better methodological quality.

## References related to Supplementary Table 2


1. Terakawa F, Kamoda H, Yonemoto T, et al. Analysis of implants for metastatic bone tumors of the proximal femur: A retrospective study. *Asia Pac J Clin Oncol*. Oct 2023;19(5):e320-e325. doi:10.1111/ajco.13921
2. Tanaka A, Okamoto M, Kito M, et al. Points of consideration when performing surgical procedures for proximal femoral bone metastasis. *J Orthop Sci*. Jan 2022;27(1):229-234. doi:10.1016/j.jos.2020.11.011
3. Vitiello R, Perisano C, Greco T, et al. Intramedullary nailing vs modular megaprosthesis in extracapsular metastases of proximal femur: clinical outcomes and complication in a retrospective study. *BMC Musculoskelet Disord*. Sep 13 2022;22(Suppl 2):1069. doi:10.1186/s12891-022-05728-5
4. Gusho CA, Clayton B, Mehta N, Hmeidan W, Colman MW, Gitelis S, Blank AT. Internal fixation versus endoprosthetic replacement of the proximal femur for metastatic bone disease: Single institutional outcomes. *J Orthop*. Nov-Dec 2021;28:86-90. doi:10.1016/j.jor.2021.11.003
5. Meynard P, Seguineau A, Laumonerie P, et al. Surgical management of proximal femoral metastasis: Fixation or hip replacement? A 309 case series. *Orthop Traumatol Surg Res*. Oct 2020;106(6):1013-1023. doi:10.1016/j.otsr.2020.05.007
6. Sørensen MS, Horstmann PF, Hindsø K, Petersen MM. Use of endoprostheses for proximal femur metastases results in a rapid rehabilitation and low risk of implant failure. A prospective population-based study. *J Bone Oncol*. Dec 2019;19:100264. doi:10.1016/j.jbo.2019.100264
7. Angelini A, Trovarelli G, Berizzi A, Pala E, Breda A, Maraldi M, Ruggieri P. Treatment of pathologic fractures of the proximal femur. *Injury*. Nov 2018;49 Suppl 3:S77-s83. doi:10.1016/j.injury.2018.09.044
8. Guzik G. Oncological and functional results after surgical treatment of bone metastases at the proximal femur. *BMC Surg*. Jan 25 2018;18(1):5. doi:10.1186/s12893-018-0336-0
9. Yu Z, Xiong, Y., Shi, R., Min, L., Zhang, W., Liu, H. ... Duan, H. Surgical management of metastatic lesions of the proximal femur with pathological fractures using intramedullary nailing or endoprosthetic replacement. . *Molecular and Clinical Oncology*. 2018;8:107-114. doi:https://doi.org/10.3892/mco.2017.1503
10. Gao H, Liu Z, Wang B, Guo A. Clinical and functional comparison of endoprosthetic replacement with intramedullary nailing for treating proximal femur metastasis. *Chin J Cancer Res*. Apr 2016;28(2):209-14. doi:10.21147/j.issn.1000-9604.2016.02.08
11. Janssen SJ, Kortlever JT, Ready JE, et al. Complications After Surgical Management of Proximal Femoral Metastasis: A Retrospective Study of 417 Patients. *J Am Acad Orthop Surg*. Jul 2016;24(7):483-94. doi:10.5435/jaaos-d-16-00043
12. Tsuda Y, Yasunaga H, Horiguchi H, Fushimi K, Kawano H, Tanaka S. Complications and Postoperative Mortality Rate After Surgery for Pathological Femur Fracture Related to Bone Metastasis: Analysis of a Nationwide Database. *Ann Surg Oncol*. Mar 2016;23(3):801-10. doi:10.1245/s10434-015-4881-9
13. Fakler JKM, Hase F, Böhme J, Josten C. Safety aspects in surgical treatment of pathological fractures of the proximal femur – modular endoprosthetic replacement vs. intramedullary nailing. *Patient Safety in Surgery*. 2013/12/07 2013;7(1):37. doi:10.1186/1754-9493-7-37
14. Weiss RJ, Ekström W, Hansen BH, et al. Pathological subtrochanteric fractures in 194 patients: a comparison of outcome after surgical treatment of pathological and non-pathological fractures. *J Surg Oncol*. Apr 2013;107(5):498-504. doi:10.1002/jso.23277
15. Harvey N, Ahlmann ER, Allison DC, Wang L, Menendez LR. Endoprostheses last longer than intramedullary devices in proximal femur metastases. *Clin Orthop Relat Res*. Mar 2012;470(3):684-91. doi:10.1007/s11999-011-2038-0
16. Steensma M, Boland PJ, Morris CD, Athanasian E, Healey JH. Endoprosthetic treatment is more durable for pathologic proximal femur fractures. *Clin Orthop Relat Res*. Mar 2012;470(3):920-6. doi:10.1007/s11999-011-2047-z
17. Parker MJ, Khan AZ, Rowlands TK. Survival after pathological fractures of the proximal femur. *Hip Int*. Sep-Oct 2011;21(5):526-30. doi:10.5301/hip.2011.8654
18. Zacherl M, Gruber G, Glehr M, et al. Surgery for pathological proximal femoral fractures, excluding femoral head and neck fractures. *International Orthopaedics*. 2011/10/01 2011;35(10):1537-1543. doi:10.1007/s00264-010-1160-z
19. Wedin R, Bauer HC. Surgical treatment of skeletal metastatic lesions of the proximal femur: endoprosthesis or reconstruction nail? *J Bone Joint Surg Br*. Dec 2005;87(12):1653-7. doi:10.1302/0301-620x.87b12.16629
